# A Web-Based Group Cognitive Behavioral Therapy Intervention for Symptoms of Anxiety and Depression Among University Students: Open-Label, Pragmatic Trial

**DOI:** 10.2196/27400

**Published:** 2021-05-27

**Authors:** Jason Bantjes, Alan E Kazdin, Pim Cuijpers, Elsie Breet, Munita Dunn-Coetzee, Charl Davids, Dan J Stein, Ronald C Kessler

**Affiliations:** 1 Institute for Life Course Health Research Department of Global Health, Faculty of Medicine and Health Sciences Stellenbosch University Stellenbosch South Africa; 2 Department of Psychology Yale University New Haven, CT United States; 3 Department of Clinical, Neuro and Developmental Psychology Faculty of Behavioural and Movement Sciences Vrije Universiteit Amsterdam Netherlands; 4 Centre for Student Counselling and Development Student Affairs Stellenbosch University Stellenbosch South Africa; 5 Department of Psychiatry and Mental Health University of Cape Town South African Medical Research Council Unit on Risk & Resilience in Mental Disorders Cape Town South Africa; 6 Department of Health Care Policy Harvard Medical School Boston, MA United States

**Keywords:** anxiety, cognitive behavioral therapy, depression, e-intervention, group therapy, web-based, university students, South Africa

## Abstract

**Background:**

Anxiety and depression are common among university students, and university counseling centers are under pressure to develop effective, novel, and sustainable interventions that engage and retain students. Group interventions delivered via the internet could be a novel and effective way to promote student mental health.

**Objective:**

We conducted a pragmatic open trial to investigate the uptake, retention, treatment response, and level of satisfaction with a remote group cognitive behavioral therapy intervention designed to reduce symptoms of anxiety and depression delivered on the web to university students during the COVID-19 pandemic.

**Methods:**

Preintervention and postintervention self-reported data on anxiety and depression were collected using the Generalized Anxiety Disorder-7 and Patient Health Questionnaire-9. Satisfaction was assessed postintervention using the Client Satisfaction with Treatment Questionnaire.

**Results:**

A total of 175 students were enrolled, 158 (90.3%) of whom initiated treatment. Among those initiating treatment, 86.1% (135/158) identified as female, and the mean age was 22.4 (SD 4.9) years. The mean number of sessions attended was 6.4 (SD 2.8) out of 10. Among participants with clinically significant symptoms at baseline, mean symptom scores decreased significantly for anxiety (t_56_=11.6; *P*<.001), depression (t_61_=7.8; *P*<.001), and composite anxiety and depression (t_60_=10.7; *P*<.001), with large effect sizes (*d*=1-1.5). Remission rates among participants with clinically significant baseline symptoms were 67.7%-78.9% and were not associated with baseline symptom severity. High overall levels of satisfaction with treatment were reported.

**Conclusions:**

The results of this study serve as a proof of concept for the use of web-based group cognitive behavioral therapy to promote the mental health of university students.

## Introduction

### Background

Depression is the most common mental disorder and the leading cause of mental health–related disease burden worldwide, affecting more than 300 million people globally and representing a major barrier to sustainable development in all regions of the world [1,2]. Depression is strongly associated with anxiety disorders. If left untreated, these disorders compromise psychosocial function and physical health and are associated with reductions in life expectancy of 11-20 years [3,4]. Both anxiety and depression are common among university students, with a 12-month prevalence for generalized anxiety disorder (GAD) and major depressive episode (MDE) of 16.7% and 18.5%, respectively [5]. In student populations, mood disorders are associated with severe role impairment [6], academic failure [7], and suicide [8]. Despite the availability of viable treatments, the majority of anxious and depressed students, like others in the general population, do not seek care [9-11]. One large cross-national study found that only 25.3%-36.3% of students with mental health problems received treatment [12]. There are a number of reasons that students do not access treatment, including a reluctance to receive help from mental health professionals [13], inability to recognize psychopathology [14], practical issues (eg, time constraints and scheduling problems), and psychological factors (eg, stigma and perceptions of therapy’s effectiveness) [15,16]. High rates of attrition among students who access campus-based treatment for anxiety and depression also contribute to the treatment gap [17].

Two key challenges faced by university student counseling centers are how to respond to the large number of students with common mental disorders and how to engage and retain students in effective treatments once they reach out for professional help [13]. Many student counseling centers are underresourced and have difficulty in reaching students in need even when they are operating at full capacity [18], with the situation being more dire in low- and middle-income countries where student counseling services are often nonexistent [19]. Self-guided and guided digital interventions are potentially scalable and cost-effective ways to close the mental health treatment gap and have been shown to be effective in treating university students with anxiety and depression [20-22], although many of these interventions have high rates of attrition and low rates of engagement [20]. Group interventions may be a more viable alternative [19], given that group therapy has been shown to be effective and to have better retention rates than digital interventions [23]. Another appeal of group interventions is that they can be offered remotely using web-based video conferencing platforms, thereby providing greater availability than traditional psychotherapy. Remote group interventions also have the potential to provide greater anonymity than conventional psychotherapy, addressing a major barrier to treatment typically faced by students [13,14]. Another appeal of web-based groups is that they can enable remote treatment when it is not possible for therapists and students to meet face to face, as was the case during the global COVID-19 pandemic [24].

### Group Cognitive Behavioral Therapy

Cognitive behavioral therapy (CBT) is a widely used evidence-based treatment for anxiety and depression [25] and can be effectively delivered via individual and group therapies [26,27], self-help interventions [28], and digital programs [29,30]. The digital revolution has precipitated the development of CBT mobile apps [31], machine learning–based chatbots that provide real-time CBT coaching [32], and guided web-based CBT group courses [33]. A meta-analysis of 35 group cognitive behavioral therapy (GCBT) clinical trials in which a protocol-based intervention that included a minimum of cognitive restructuring and behavioral activation was delivered face to face to adults in 5-16 sessions concluded that GCBT is an effective alternative to individual therapy and compares favorably with other middle-intensity interventions [27]. Evidence also exists that GCBT is effective among students both as a treatment and as an indicated prevention for anxiety and depression [34-36] and that such group therapies can be delivered effectively on the web [33]. Indeed, web-based GCBT might be particularly appropriate for university students given the openness of students to digital interventions and the potential of this modality to overcome barriers to care [37,38], although evidence is needed to show that GCBT is effective and satisfies students’ needs.

For web-based GCBT to be meaningfully integrated into student counseling services, it is necessary to not only establish that this modality is effective but also understand which students respond well to it. Adopting a precision medicine (personalized medicine) approach to predict treatment effects is integral to designing evidence-based, efficient, and effective student mental health services [39]. In addition, web-based GCBT could be integrated into stepped care treatments if we understand how symptom severity predicts treatment response [40]. Identifying predictors of treatment response to GCBT could also aid the development of machine learning prediction models that use patient-level data to individualize student mental health care [41]. Predictors of treatment response for traditional interventions for mood disorders include sociodemographic variables (eg, age and sex) and clinical factors (eg, symptom profiles, comorbidities, and symptom severity) [41-43].

Before attempting to conduct controlled trials to document aggregate treatment effects for web-based GCBT or studies designed to develop precision treatment algorithms, it is important to establish whether this modality would engage students and satisfy their needs. Ensuring satisfaction with treatment is an integral component of engaging and retaining students in metal health interventions, and it enables a person-centered approach to student wellness services [44]. Evidence suggests that students are satisfied with web-based CBT interventions irrespective of whether they are delivered by therapist or are self-administered [45], but it remains unclear whether web-based GCBT could satisfy students’ needs.

### Objectives

The aim of this study is to present the results of a pragmatic open trial to address these uncertainties. We investigate the uptake, attendance, treatment responses, predictors of treatment response, and satisfaction with a 10-session web-based GCBT intervention for anxiety and depression. This pragmatic trial was implemented in South Africa during the global COVID-19 pandemic, when access to traditional campus-based psychotherapy was restricted. A pragmatic design was used for this trial because we wanted to establish whether web-based GCBT could be implemented under real-world conditions to promote mental health in a broad population of students [46]. Unlike explanatory trials that seek to test whether interventions work under optimal conditions with a clearly defined population, pragmatic trials evaluate intervention effects in routine practice conditions [47]. This research is a part of the ongoing work to identify effective and sustainable interventions to promote student mental health by the World Health Organization World Mental Health Surveys International College Student Initiative [48].

## Methods

### Recruitment

Information about the intervention was posted only once on a student affairs Facebook page at Stellenbosch University in South Africa (N=32,600 students, approximately) near the start of the COVID-19 pandemic when South Africa was in lockdown and access to campus-based counseling was restricted. The posting explained that web-based groups were being offered to help students learn psychological skills to reduce symptoms of anxiety and depression. Although it is not possible to ascertain how many students would have seen the post or how many students who did see it would have shared the information with friends, we know that this Facebook page was followed by 3344 people at the time of recruitment. The 175 students who responded within 24 hours to the notice completed a baseline assessment and provided informed consent before being randomized across 15 groups (to keep group sizes to between 10 and 15 participants). No student who wanted to participate was excluded, and no incentives were given to participate.

### Group Content

The intervention was delivered via Microsoft Teams (a secure web-based video conferencing platform) in 10 weekly workshops of 60-75 minutes. The content was drawn from common elements identified from GCBT interventions that were shown to be effective for university students [35,49-52]. The content (Textbox 1) was organized into 5 themes, with each theme spanning 2 workshops. To make the intervention more engaging and relevant to the target population, we consulted a group of 4 student advisors regularly over the development period to select suitable examples, plan activities, and inform the layout and design of materials. Consultation with the student advisors took place in an iterative process over the course of a year while we developed and refined the intervention materials. We also consulted 3 psychologists working in student counseling centers, and the intervention materials were critically reviewed by 2 CBT experts.

Participants were provided with electronic interactive PDF workbooks (Adobe Acrobat) consisting of exercises and brief summaries of the main ideas and skills for each session before each workshop. Participants were given the option to remain anonymous by keeping their web cameras off and/or by using pseudonyms, although they were encouraged to use web cameras to show their faces during the workshops. Participants were also invited to use the web-based chat function to type comments, questions, or responses during the sessions if they felt uncomfortable speaking in the group. The intervention was offered in partnership with the student counseling center and was positioned as a university-endorsed program integrated into the routine clinical care offered to students. Strategies used to improve retention included giving participants permission to miss sessions but encouraging attendance at each new session, sending follow-up emails to students who missed sessions prompting them to join the following week, and giving a brief recap of the previous workshop at the start of each new session.

Sessions were facilitated by registered counselors (the equivalent of psychological technicians with 4 years of university training) and clinical psychology master’s students under the supervision of a registered PhD psychologist. A facilitators’ handbook, with detailed descriptions of the content of each session and facilitation guidelines, was provided, and facilitators were trained on group therapy facilitation. Web-based sessions were recorded so that they could be reviewed under supervision.

**Overview of the intervention content.**

**Theme 1: You feel the way you think**

- Workshop 1: Emotional triggers and automatic thoughts
  How to identify activating events (emotional triggers) and recognize how automatic thoughts contribute to the way you feel
- Workshop 2: Challenging automatic thoughts and core beliefs
  How to identify and challenge unhelpful core beliefs and automatic thoughts

**Theme 2: Planning to succeed**

- Workshop 1: Getting on top of problems before they get you down
  How to recognize stressors and use strategies to solve interpersonal and emotional problems
- Workshop 2: Goal setting and planning
  How to set goals and plan for behavior change

**Theme 3: Hacks to boost your mood**

- Workshop 1: Avoiding thinking traps
  How to identify and modify dysfunctional patterns of thinking
- Workshop 2: Overcoming rumination and guilt
  How to use strategies to overcome rumination and guilt

**Theme 4: Building mastery**

- Workshop 1: Behavior activation
  How to identify and increase activities that promote feelings of well-being and reduce stress
- Workshop 2: Behaviors that matter
  How to identify unhealthy habits and develop health-promoting behaviors

**Theme 5: Avoiding meltdowns**

- Workshop 1: Understanding the body’s stress response
  Understanding the body’s stress response and how to use strategies to regulate physiological arousal
- Workshop 2: Managing stress and overcoming avoidance
  How to manage stress and overcome avoidance

### Measures

Self-reported preintervention and postintervention surveys were administered on the web using Qualtrics software to record the following sociodemographic and clinical characteristics of participants.

#### Demographics

In the preintervention survey, participants were asked about their self-identified population group, gender, age, and current year of registration.

#### Symptoms of Anxiety and Depression

The Generalized Anxiety Disorder-7 (GAD-7) scale [53] and Patient Health Questionnaire-9 (PHQ-9) [54] were administered preintervention and 1-week postintervention. The GAD-7 consists of 7 items assessing the frequency of core symptoms of anxiety disorders over the past 2 weeks, scored using a response scale from 0 (not at all) to 3 (nearly every day) and yielding a total score ranging from 0 to 21. A cutoff of 10 is used to identify clinically significant symptoms (ie, individuals likely to meet the diagnostic criteria for GAD) [53]. Psychometric studies have documented good GAD-7 internal consistency reliability, validity, and sensitivity to changes in the severity of anxiety symptoms in a range of settings, including in Africa [55,56]. The PHQ-9 is a self-reported instrument consisting of 9 items assessing the frequency of core symptoms of an MDE over the past 2 weeks on a 0-4 response scale, yielding a total score ranging from 0 to 27. A cutoff of 10 is used to identify clinically significant symptoms (ie, individuals likely to meet the diagnostic criteria for MDE) [53,57]. The PHQ-9 has good internal consistency reliability, validity, and sensitivity to changes in the severity of depressive symptoms in a range of settings [58,59], including in South Africa [60]. Cutoffs of 5, 10, and 20 on the GAD-7 and PHQ-9 were used to identify individuals with none, mild, moderate, or severe symptoms.

#### Client Satisfaction With Treatment

The postintervention survey included the Client Satisfaction with Treatment Questionnaire-8, an 8-item self-reported questionnaire with high internal consistency reliability [61] that provides an efficient, sensitive, and reasonably comprehensive measure of patient satisfaction with services [61,62].

The primary outcomes were symptoms of anxiety (ie, GAD-7 scores), symptoms of depression (ie, PHQ-9 scores), and symptoms of comorbid anxiety and depression (using combined GAD-7 and PHQ-9 scores as proposed by the developers of the instruments to yield a composite anxiety and depression score) [63].

### Data Analysis

The data were cleaned and analyzed using SPSS, version 27 (IBM Corporation). Descriptive statistics were used to summarize participant characteristics, attendance rates, primary outcomes, and satisfaction with treatment. A repeated-measures analysis of variance (ANOVA) was used to measure changes in symptoms scores from baseline to follow-up, with effect size determined by η^2^. The mean symptom scores at baseline and follow-up were also compared using a paired-sample *t* test, with effect sizes determined by Cohen *d* calculated using Comprehensive Meta-Analysis (CMA) software and the procedures described by Borenstein et al [64]. The McNemar test was used to evaluate the significance of changes in proportions, and multiple regression analysis was used to identify predictors of treatment response and satisfaction with treatment. The analysis was conducted on the subsample of all participants who started the intervention, irrespective of whether they completed the intervention (ie, an intention-to-treat analysis). Participants who did not complete the follow-up assessments were excluded from the analysis via listwise deletion. The results of all regression analyses are reported as adjusted odds ratios (aORs) for logistic regression models and standardized partial regression coefficients for linear regression models, with 95% CI. Statistical significance was evaluated using *P*=.05-level 2-sided tests.

### Ethical Considerations

Ethical approval was obtained from the Health Science Research Ethics Committee (N19/10/145), and institutional permission was secured before recruitment. Informed consent was obtained electronically. The participants were given an opportunity to participate in the intervention without being enrolled in the study, although none of them opted for this arrangement. The deidentified data were stored on a password-protected computer. All participants were provided with contact details of the 24-hour campus crisis service, and students who reported suicidal ideation were contacted via email to prompt them to seek individual care.

## Results

### Sample Characteristics

**Figure 1:**
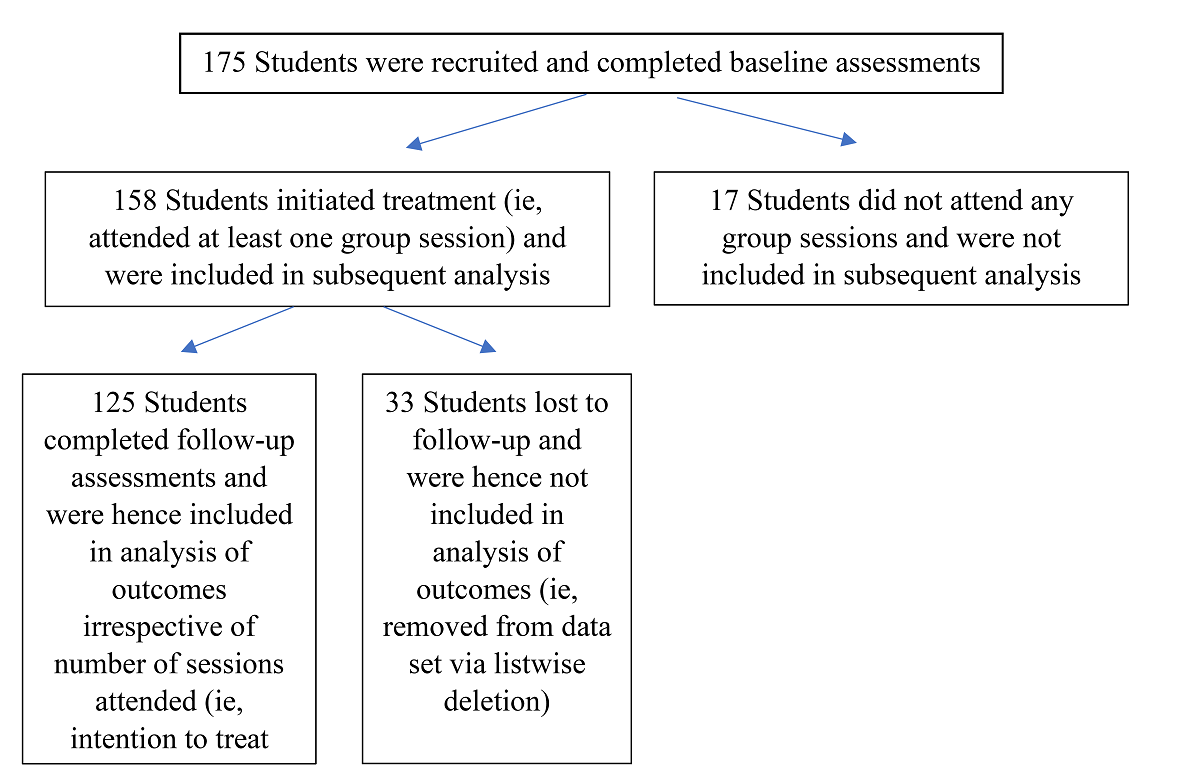
Flowchart of recruitment and assessment process.

Of the 175 students who were enrolled and completed baseline assessments, 158 (90.3%) initiated treatment and 125 (71.4%) completed follow-up assessments (Figure 1). No significant baseline differences were found between participants who initiated treatment and those who did not in terms of gender (*P*=.30), age (*P*=.76), undergraduate status (*P*=.24), or baseline severity of anxiety (*P*=.72) or depression (*P*=.06). Participants who were lost to follow-up were not significantly different from participants who were followed up with respect to gender (*P*=.91), age (*P*=.29), undergraduate status (*P*=.16), or baseline severity of anxiety (*P*=.24) or depression (*P*=.27; see Tables S1-S4 in Multimedia Appendix 1). However, attendance was lower among participants who were lost to follow-up (mean number of sessions 4.8 vs 6.8; *P*=.02).

The mean age of the sample of students for whom treatment was initiated (n=158) was 22.2 (SD 4.9) years, with a range of 18-54 years (median 21 years, mode 19 years). Among them, 75.9% (120/158) were undergraduates and 24.1% (38/158) were postgraduates. Overall, 85.4% (135/158) self-identified as female, 13.9% (22/158) as male, and 1 (0.6%) student identified as gender nonbinary. Of these, 20.9% (33/158) self-identified as Black African, 19.6% (31/158) as colored (an official term used in South Africa for population classification), 7% (11/158) as Asian, and 52.5% (83/158) as White. The mean baseline GAD-7 and PHQ-9 scores were 9.5 (SD 5.5) and 10.2 (SD 5.4), with 21.5% (34/158) of students scoring less than 5 on the GAD-7 and 14.6% (23/158) scoring less than 5 on the PHQ-9 preintervention survey. The mean number of sessions attended was 6.4 (SD 2.8), with 72.8% (115/158) of participants attending half or more of the sessions and 46.2% (73/158) attending 80% or more (Table 1). Only 10.1% (16/158) of participants attended all 10 sessions. The reasons for nonattendance included competing academic commitments, internet connectivity problems, power failures, and insufficient internet data.

**Table 1:** Frequency and rate of attendance by number of sessions (n=158).

Number of sessions attended	Participants, n (%)	Participants (cumulative), n (%)
10	16 (10.1)	16 (10.1)
9	31 (19.6)	47 (29.7)
8	26 (16.5)	73 (46.2)
7	21 (13.3)	94 (59.5)
6	13 (8.2)	107 (67.7)
5	8 (5.1)	115 (72.8)
4	7 (4.4)	122 (77.2)
3	14 (8.9)	136 (86.1)
2	8 (5.1)	144 (91.1)
1	14 (8.9)	158 (100)

### Improvements in Aggregate Symptoms Scores

To assess intervention effect sizes, we conducted a repeated measures ANOVA comparing symptom scores from baseline to follow-up and calculated the changes in mean symptom scores from baseline to follow-up (with associated effect sizes) for anxiety, depression, and composite anxiety and depression (Table 2). To establish if the effect sizes varied according to symptom severity, we investigated changes in mean scores among the subset of participants with clinically significant symptoms and among subsets with mild, moderate, and severe symptoms. The repeated measures ANOVA showed significant symptom reductions for anxiety (*F*_1,124_=66.2; *P*<.001) and depression (*F*_1,123_=45.4; *P*<.001), with large effect sizes for anxiety (η^2^=0.4) and depression (η^2^=0.27). Among participants with clinically significant symptoms at baseline, the mean symptom scores decreased significantly for anxiety (t_56_=11.6; *P*<.001), depression (t_61_=7.8; *P*<.001), and composite anxiety and depression (t_60_=10.7; *P*<.001), with large effect sizes (*d*=1-1.5).

**Table 2:** Comparison of mean symptom scores at baseline and follow-up (paired-sample *t* test with effect size; n=125).

Symptom severity			Baseline, mean (SD)		Follow-up, mean (SD)		*t* test *(df)*		*P* value		Correlation		Cohen *d*					Effect size
													*d*		SE			
**Symptoms of anxiety (GAD^a^-7 scores)**																		
	Clinically significant symptoms^b^	14.1 (3.5)		6.9 (4.2)		11.6 (56)		<.001^c^		0.3		1.9		0.3		Large		
	Mild symptoms^d^	7.3 (1.5)		5.6 (4.3)		2.3 (36)		.03^c^		0.1		0.5		0.2		Medium		
	Moderate symptoms^e^	13.5 (3.1)		6.5 (3.7)		11.2 (51)		<.001		0.2		2.0		0.3		Large		
	Severe symptoms^f^	20.2 (0.4)		11.0 (6.9)		3.0 (4)		.04^c^		0.5		1.4		0.6		Large		
**Symptoms of depression (PHQ-9^g^** **scores)**																		
	Clinically significant symptoms	14.0 (3.6)		8.5 (5.0)		7.8 (61)		<.001		0.2		1.3		0.2		Large		
	Mild symptoms	7.1 (1.3)		4.8 (3.1)		4.8 (42)		<.001		0.2		0.9		0.2		Large		
	Moderate symptoms	13.2 (2.4)		8.1 (4.6)		7.6 (56)		<.001		0.1		1.4		0.3		Large		
	Severe symptoms	23.0 (2.3)		12.4 (7.8)		2.6 (4)		.06		−0.5		2.0		1.4		Large		
**Symptoms of anxiety and depression (GAD-7+PHQ-9 scores)**																		
	Clinically significant symptoms^h^	27.2 (5.7)		15.4 (7.7)		10.7 (60)		<.001		0.2		1.7		0.3		Large		
	Mild symptoms^i^	14.5 (2.7)		10.0 (9.2)		3.1 (38)		<.001		0.2		0.6		0.2		Medium		
	Moderate symptoms^j^	26.7 (5.1)		15.0 (7.0)		10.8 (58)		<.001		0.1		1.9		0.3		Large		
	Severe symptoms^k^	41.5 (0.7)		28.0 (19.8)		0.9 (1)		.53		−1.0		0.1		0.1		Small		

^a^GAD: generalized anxiety disorder.

^b^Generalized Anxiety Disorder-7/Patient Health Questionnaire-9 score ≥10.

^c^*P*<.05.

^d^Patient Health Questionnaire-9/Generalized Anxiety Disorder-7 score 5-9.

^e^Patient Health Questionnaire-9/Generalized Anxiety Disorder-7 score 10-19.

^f^Patient Health Questionnaire-9/Generalized Anxiety Disorder-7 score ≥20.

^g^PHQ-9: Patient Health Questionnaire-9.

^h^Patient Health Questionnaire-9+Generalized Anxiety Disorder-7 score ≥20.

^i^Patient Health Questionnaire-9+Generalized Anxiety Disorder-7 10-19.

^j^Patient Health Questionnaire-9+Generalized Anxiety Disorder-7 20-39.

^k^Patient Health Questionnaire-9+Generalized Anxiety Disorder-7 score ≥40.

The mean symptom scores decreased comparably for participants with moderate and severe symptoms at baseline. The mean symptom scores also decreased among participants with mild symptoms of anxiety (t_36_=2.3; *P*=.03), depression (t_42_=4.8; *P*<.001), and the anxiety and depression composite (t_38_=3.1; *P*<.001) at baseline, with a large effect size for depression (*d*=0.9) and medium effect sizes for anxiety (*d*=0.5) and composite anxiety depression (*d*=0.6). The mean symptom scores decreased significantly, irrespective of symptom severity at baseline, although the effects were largest for moderate and severe symptoms (Table 2).

### Treatment Response and Remission Rates

The proportion of participants with clinically significant symptoms decreased significantly from 45.6% (SE 4.5) to 16.9% (SE 3.4) for anxiety (χ^2^_1_=22.7; *P*<.001), from 49.6% (SE 4.5) to 8.4% (SE 3.5) for depression (*χ*^2^_1_=22.7-32; *P*<.001), and from 48.8% (SE 4.5) to 15.2% (SE 3.2) for the anxiety and depression composite (*χ*^2^_1_=33.6; *P*<.001). These changes represent 54.1%-68.9% proportional reductions in prevalence. The response and remission rates were calculated across all levels of symptom severity to demonstrate how many participants reported changes in their symptom profiles (Table 3), showing significant changes for anxiety (*χ*^2^_3_=26.3; *P*<.001), depression (*χ*^2^_3_=30.6; *P*<.001), and composite anxiety and depression (*χ*^2^_3_=35.3; *P*<.001).

**Table 3:** Prevalence of symptoms at baseline and follow-up (n=125).

Symptoms	No symptoms^a^, % (SE)	Mild symptoms^b^, % (SE)	Moderate symptoms^c^, % (SE)	Severe symptoms^d^, % (SE)	*χ*^2^ (*df*)	*P* value
Symptoms of anxiety^e^ at baseline	24.8 (3.9)	29.6 (4.1)	25.6 (3.9)	20.0 (3.6)	N/A^f^	N/A
Symptoms of anxiety^e^ at follow-up	46.8 (4.5)	36.3 (4.3)	10.5 (2.8)	6.5 (2.2)	26.3 (3)	<.001^g^
Symptoms of depression^h^ at baseline	16.0 (3.3)	34.4 (4.3)	45.6 (4.5)	4.0 (1.8)	N/A	N/A
Symptoms of depression^h^ at follow-up	37.6 (4.3)	44.0 (4.5)	16.0 (3.3)	2.4 (1.4)	30.6 (3)	<.001
Composite anxiety and depression^i^ at baseline	20.0 (3.6)	31.2 (4.2)	29.6 (4.1)	19.2 (3.5)	N/A	N/A
Composite anxiety and depression^i^ at follow-up	43.2 (4.4)	41.6 (4.4)	11.2 (2.8)	4.0 (1.8)	35.3 (3)	<.001

^a^Patient Health Questionnaire-9/Generalized Anxiety Disorder-7 score <5.

^b^Patient Health Questionnaire-9/Generalized Anxiety Disorder-7 score 5-9.

^c^Patient Health Questionnaire-9/Generalized Anxiety Disorder-7 score 10-19.

^d^Patient Health Questionnaire-9/Generalized Anxiety Disorder-7 score ≤20.

^e^Generalized Anxiety Disorder-7 score.

^f^N/A: not applicable.

^g^*P*<.05.

^h^Patient Health Questionnaire-9 score.

^i^Patient Health Questionnaire-9+Generalized Anxiety Disorder-7 score.

The proportion of participants with severe symptoms (ie, scores of 20 or more on the GAD-7 or PHQ-9), reduced significantly among those with symptoms of anxiety (20.0% vs 6.5%; *P*<.001), depression (4.0% vs 2.4%; *P*<.001), and composite anxiety and depression (19.2% vs 4.0%; *P*<.001). The proportions of participants with moderate symptoms (ie, scores of 10-19 on the GAD-7 or PHQ-9) decreased significantly for anxiety (25.6% vs 10.5%; *P*<.001), depression (45.6% vs 6.0%; *P*<.001) and composite anxiety and depression (29.6% vs 11.2%; *P*<.001). As expected, the proportion of patients with mild symptoms increased from between 28.8% and 34.4% at baseline to 36.3% and 44.0% across all outcomes at follow-up, and the proportion of asymptomatic patients increased from 12%-24.8% at baseline to 32.0%-46.8% across all outcomes at follow-up.

To provide a more nuanced perspective on response to treatment, we calculated rates of remission (defined as a reduction in GAD-7/PHQ-9 scores below 10), treatment response (defined as a 50% or greater reduction in baseline symptom scores), and clinical deterioration (defined as a 50% or greater increase in baseline symptom scores; Table 4). Among participants reporting clinically significant baseline symptoms, remission was in the range 67.7%-78.9% across outcomes, and clinically significant deterioration was uncommon, ranging from 1.8% to 3.2% across outcomes. Changes were also observed among participants who at baseline had mild symptoms, among whom treatment response was in the range of 34.9%-43.6% across outcomes and clinically significant deterioration was in the range of 4.7%-10.8% across outcomes. The data indicate the potential for the intervention to reduce the absolute number of students with clinically significant symptoms and demonstrate the low numbers expected to deteriorate over the course of the intervention.

**Table 4:** Individual-level response (n=125).

Symptoms			Among participants with clinically significant symptoms at baseline^a^				Among participants with mild symptoms at baseline		
			Remission^b^		Deterioration^c^		Treatment response^d^		Deterioration
**Symptoms of anxiety^e^**									
	% (SE)	67.7 (6.0)		3.2 (2.3)		34.9 (7.4)		4.7 (3.2)	
	n (%)	62 (49.6)		62 (49.6)		43 (34.4)		43 (34.4)	
**Symptoms of depression^f^**									
	% (SE)	78.9 (5.4)		1.8 (1.8)		35.1 (8.0)		10.8 (5.2)	
	n (%)	57 (45.6)		57 (45.6)		37 (29.6)		37 (29.6)	
**Composite anxiety and depression^g^**									
	% (SE)	75.4 (5.6)		3.3 (1.6)		43.6 (8.0)		7.7 (4.3)	
	n (%)	61 (48.8)		61 (48.8)		39 (31.2)		39 (31.2)	

^a^Generalized Anxiety Disorder-7/Patient Health Questionnaire-9 score ≥10.

^b^Remission: reduction of symptoms so that Patient Health Questionnaire-9/Generalized Anxiety Disorder-7 score <10.

^c^Deterioration: a 50% increase in symptoms and a change from no to mild symptoms, from mild to moderate symptoms, or from moderate to severe symptoms.

^d^Treatment response: a reduction in symptoms of 50% or more.

^e^Generalized Anxiety Disorder-7 score ≥10.

^f^Patient Health Questionnaire-9 score ≥10.

^g^Patient Health Questionnaire-9+Generalized Anxiety Disorder-7 ≥20.

### Predictors of Treatment Response and Remission

We investigated whether information collected from participants at baseline could be used to identify individuals who were likely to respond to the intervention. Multiple logistic regression analysis was used to identify baseline predictors of remission among participants with clinically significant symptoms at baseline and among participants with only mild baseline symptoms (Table 5). Predictors of treatment response among participants with clinically significant symptoms were not examined because of the rarity of response without remission, and predictors of symptom deterioration were not examined because of the small number of participants who deteriorated.

Among participants with clinically significant baseline symptoms, baseline variables were significant in predicting remission from composite anxiety and depression (*χ*^2^_5_=17.1; *P*=.004) but not in predicting remission from anxiety (*χ*^2^_5_=9.3; *P*=.10) or depression (*χ*^2^_5_=8.8; *P*=.12). Among participants with clinically significant composite anxiety and depression, identifying as female was associated with substantially increased odds of remission (aOR 9.6, 95% CI 1.2-78.5; *P*=.04), whereas baseline depression symptom severity was associated with a modestly decreased odds of remission (aOR 0.8, 95% CI 0.7-1; *P*=.03). No other baseline variables were associated with remission among the participants with clinically significant symptoms. It is noteworthy that the substantial aOR associated with female sex predicting remission from anxiety was, to some extent, because of the comparatively small proportion of male participants, as an investigation of zero-order before-and-after changes in prevalence found little evidence of differences between women (44.9% before to 15.0% after) and men (50.0% before to 27.8% after; Tables S5 and S6 in Multimedia Appendix 1).

Among participants with mild baseline symptoms, baseline variables were significant in predicting treatment response for anxiety (*χ*^2^_5_=12.3; *P*=.03) but not in predicting treatment response for depression (*χ*^2^_5_=5.8; *P*=.33) or composite anxiety and depression (*χ*^2^_5_=5.6; *P*=.35). The only variable associated with treatment response was baseline depression symptom severity, which was associated with decreased odds of treatment response for anxiety (aOR 0.6, 95% CI 0.4-0.9; *P*=.02).

**Table 5:** Predictors of treatment response (n=125).

Symptoms			Female sex					Age					Number of sessions					Baseline PHQ^a^					Baseline GAD^b^					*χ*^2^ (*df*)			*P* value
			OR^c^ (95% CI)		*P* value		OR (95% CI)			*P* value		OR (95% CI)			*P* value		OR (95% CI)			*P* value		OR (95% CI)			*P* value						
**Remission^d^** **among participants with clinically significant symptoms at baseline**																															
	Symptoms of anxiety^e^, (n=57)	8.1 (1.0-68.0)		.05		1.1 (0.9-1.4)			.43		1.3 (0.9-1.7)			.11		0.9 (0.8-1.1)			.29		0.9 (0.7-1.1)			.16		9.3 (5)			.10		
	Symptoms of depression^f^, (n=62)	1.9 (0.3-11.5)		.49		1.1 (0.9-1.4)			.21		1.1 (0.9-1.4)			.32		0.9 (0.8-1)			.11		1.1 (0.9-1.3)			.14		8.8 (5)			.12		
	Composite anxiety and depression^g^, (n=61)	9.6^h^ (1.2-78.5)		.03^h^		1.3 (0.9-1.8)			.07		1.2 (0.9-1.6)			.13		0.8^h^ (0.7-1)			.03		1.1 (0.9-1.3)			.28		17.1 (5)			.004^h^		
**Treatment response^i^** **among participants with mild symptoms at baseline**																															
	Symptoms of anxiety, (n=37)	0.2 (0.0-2.2)		.19		1.0 (0.7-1.3)			.89		0.8 (0.5-1.1)			.19		0.6^h^ (0.4-0.9)			.02^h^		2.1 (1.0-4.3)			.06		12.3 (5)			.03		
	Symptoms of depression, (n=43)	2.6 (0.4-19.0)		.34		0.9 (0.7-1.1)			.21		0.8 (0.6-1.1)			.14		0.9 (0.5-1.6)			.78		1.1 (0.9-1.4)			.28		5.8 (5)			.33		
	Composite anxiety and depression, (n=39)	2.4 (0.3-20.4)		.42		0.9 (0.8-1.1)			.58		0.9 (0.6-1.2)			.33		0.7 (0.5-1.1)			.10		1.2 (0.8-1.6)			.37		5.6 (5)			.35		

^a^PHQ: Patient Health Questionnaire.

^b^GAD: generalized anxiety disorder.

^c^OR: odds ratio.

^d^Remission: reduction of symptoms so that Patient Health Questionnaire-9/Generalized Anxiety Disorder-7 score <10.

^e^Generalized Anxiety Disorder-7 score.

^f^Patient Health Questionnaire-9 score.

^g^Patient Health Questionnaire-9+Generalized Anxiety Disorder-7.

^h^*P*<.05.

^i^Treatment response: a reduction in symptoms of 50% or more.

### Satisfaction With Treatment

Most of the participants (n=125) rated intervention quality as good or excellent (114/125, 91.1%), were satisfied with the kind (108/125, 86.1%) and amount (108/125, 86.4%) of help received, reported being better able to deal effectively with their problems following the intervention (112/125, 89.6%), felt that the intervention met all or most of their needs (93/125, 74.4%), said that they would recommend the intervention to friends (119/125, 95.2%), and were satisfied overall with the intervention (113/125, 90.4%; Table S7 in Multimedia Appendix 1). Multiple linear regression analysis was used to identify baseline predictors of total satisfaction with treatment (Multimedia Appendix 1, Table S7). Female participants reported significantly higher levels of satisfaction than male participants (β=2; 95% CI 0.6-4.7; *P*=.01; Multimedia Appendix 1, Table S8). Interestingly, satisfaction was not associated with symptom improvement (β=−.1; 95% CI −1.3 to 0.7; *P*=.57), indicating that participants who showed less improvement were as satisfied with the intervention as those who showed higher levels of improvement (Multimedia Appendix 1, Table S8).

## Discussion

### Principal Findings

Students participating in this 10-week web-based intervention reported significant reductions in anxiety and depression symptom scores, with large effect sizes. Furthermore, there was a significant reduction in the proportion of participants with clinically significant symptoms from baseline to follow-up, with improvements across all levels of symptom severity. Participants reported high levels of satisfaction. This trial, which was conducted under real-world conditions in response to a global crisis, serves as a proof of concept for the use of web-based GCBT, showing that it is possible to recruit, retain, and improve the outcomes of university students with web-based GCBT.

It is noteworthy that our structured GCBT intervention was delivered by graduate clinical psychology students (ie, trainee psychologists) and counselors (with 4 years of training). This group intervention enabled us to reach a larger number of students than would otherwise have been the case using the same resources to deliver individual therapy. These findings speak to the sustainability and cost-effectiveness of the intervention, making it potentially well suited for resource-constrained environments such as South Africa, where the 12-month prevalence of common mental disorders among university students is as high as 31.5% [65] and where even at the most well-resourced institutions, only 28.9% of students with mental disorders receive treatment [19]. Session-by-session fiscal analysis has consistently shown that group therapy is less costly than individual therapy, highlighting the potential for group interventions to be scalable [66]. Efforts to expand the use of group therapy have hitherto been hampered by clinicians’ concerns that groups may not be as effective as individual psychotherapy [66], which appears not to be the case for our intervention. Subsequent studies should establish how treatment outcomes are affected by the level of facilitator training and whether the intervention could be delivered by nonprofessionals within a peer-to-peer model. Consistent with the latter possibility, evidence suggests that peer-to-peer interventions can be effective in promoting student mental health [67,68]. Peer-to-peer interventions might also be appropriate given students’ reluctance to seek help from mental health professionals [13].

Although attendance rates were relatively good, with a mean attendance of 6 sessions, only 10.1% of the students attended all sessions. This pattern is typical for students. For example, a trial of a 12-session group dialectical behavior therapy intervention for university students reported a 30.0% dropout rate with a mean attendance rate of approximately 60.0%. One survey of counseling center utilization over a 5-year period at a large US university reported that the average number of counseling sessions attended was 4 (SD 3.8, median 3, mode 1), with 18.0% of treatment seekers attending only 1 session [69]. In another study, 51.7% of students who accessed counseling attended 5 or fewer sessions [70]. However, crucially, our retention rates are markedly higher than those typically reported in self-guided or guided individualized internet-based interventions with students [71], suggesting that web-based GCBT might be more engaging than other digital interventions. The use of simple strategies (such as a flexible approach to attendance, encouraging participants to return after they miss a session, and ensuring sufficient repetition and continuity so that missing sessions does not disrupt care) seems to have been effective in retaining students, although it would be valuable to experiment with alternative retention strategies in future implementations.

The remission rates found here (67.7%-78.9%) compare favorably with those reported in clinical trials for other treatments of anxiety and depression. For example, trials of antidepressant treatment typically report response rates in the order of 70%-80% [72], and a trial of GCBT and duloxetine for GAD reported a remission rate of 21.5% [73]. The marked improvements we observed in symptom scores at an aggregate level are particularly remarkable given that we did not exclude students with mild or moderate symptoms, as would typically be the case in controlled trials [46]. The fact that individuals with mild and moderate symptoms showed significant improvements is important because this population also seeks treatment and is at risk for eventually developing clinical disorders.

Notably, it is possible that our treatment outcomes are strong because we recruited participants into this intervention during the hard lockdown in South Africa at the start of the COVID-19 pandemic, and restrictions were starting to ease as participants completed the intervention. It is also important to note that we only recruited for 24 hours, which means that the intervention took only students very eager to obtain help. By only including very eager students, we have biased our outcomes by including only highly motivated and responsive participants. Before-and-after differences in symptoms might be smaller for more representative samples of students. Nonetheless, our ability to respond quickly to a need for services and to accommodate so many students is a clear strength of this intervention. It is also noteworthy that easing of restrictions on movement occurred over the period of the intervention, undoubtedly contributing to reduced levels of anxiety and depression, although the country was still in a state of national emergency and students were still making stressful transitions to web-based learning and assessment when follow-up data were collected at the end of the intervention. It is also possible that the positive effects we observed for mean symptom scores are a result of statistical regression to the mean (ie, the tendency of extreme measures to move closer to the mean overtime). However, this is less likely than would normally be the case in clinical trials, given that we recruited participants with a wide range of symptoms, including some students with minimal symptom scores at baseline. It will be important to establish in future replications if these favorable outcomes are also observed when students are not confined to their homes and when they have access to other therapies. To this end, follow-up studies including rigorous controlled trials are needed to test the effectiveness of the intervention against other standard treatments.

Although the good outcomes we observed suggest that web-based groups may be an effective and sustainable way to increase students’ access to mental health services, it is important to remember that there are significant barriers to providing internet-based services that make them inaccessible to some students. These barriers include the need for students to have appropriate technology, access to broadband internet services and adequate data, a stable internet connection, uninterrupted power supply, and a private space in which to participate [74]. Many of these barriers will be particularly marked for students with limited financial resources, and providing web-based services may thus further exacerbate the inequality in access to treatments in low- and middle-income countries [19].

We found high levels of satisfaction with the intervention, highlighting the potential for web-based GCBT to meet the needs of anxious and depressed students. It is significant that most participants rated the quality of the intervention as good or excellent and said that they were satisfied with the amount and kind of help they received. This finding is consistent with studies reporting students’ positive attitudes toward internet-based mental health interventions [75] and provides further evidence to support the use of digital interventions to promote student wellness [38]. However, it would be helpful if subsequent studies in this area could collect more detailed qualitative data to provide a rich description of students’ lived experience of participating in web-based interventions of this kind.

It will be important for future studies in this area to shed more light on factors that predict a good treatment response to these kinds of interventions. Given that this is a group intervention, it also seems appropriate to investigate how group membership and group dynamics (such as group cohesion) may influence treatment outcomes.

### Limitations

There are 2 limitations that are worth noting. First, the study did not include a control group, as would be the case in a randomized controlled trial. As a result, we cannot infer that the observed symptom improvements were a result of the intervention. It is well established that even control groups (with no interventions) show improvements over time [76]. However, it was not our intention to undertake a controlled trial; instead, our challenge was to devise a treatment delivered in a novel digital format and tested in a real-world setting where treatment could be applied on a larger scale than is possible in individual therapy. Meeting these challenges along with the positive treatment results makes a randomized controlled trial a logical next step, as our findings clearly demonstrated that such a trial is both warranted and feasible.

Second, a relatively high number of participants dropped out, with 28.4% (45/158) of participants attending fewer than half of the sessions. Dropout rates in psychotherapy research vary widely, ranging from 0.0% to 83.0% for e-interventions [77], and it is not clear how dropping out should be interpreted, given that many clients show gains early in treatment, which may contribute to *premature* termination. Despite these limitations, our study is an important initial step toward establishing that GCBT can be effectively delivered on the web to students under real-world conditions.

### Conclusions

In conclusion, our results suggest that web-based GCBT holds promise as an effective and sustainable intervention for anxiety and depression and that university students participating in this intervention are satisfied with this modality. We demonstrated that it is possible as part of routine care in university counseling centers to recruit and retain students in web-based GCBT and deliver a remote intervention via video conferencing under real-world conditions. This novel intervention could have important implications for increasing access to psychotherapy in low- and middle-income countries. These findings indicate that larger-scale controlled trials of this modality are warranted, particularly trials that expand recruitment to include a wider range of students other than those most eager to participate, especially reaching out for more male students; investigate how retention can be improved; and examine factors that predict treatment response.

## Appendix

Multimedia Appendix 1 Detailed statistical analysis to support the findings.

## Abbreviations

ANOVA: analysis of variance
aOR: adjusted odds ratio
CBT: cognitive behavioral therapy
GAD: generalized anxiety disorder
GAD-7: Generalized Anxiety Disorder-7
GCBT: group cognitive behavioral therapy
MDE: major depressive episode
PHQ-9: Patient Health Questionnaire-9
