# Supplementary material for: A Web-Based Group Cognitive Behavioral Therapy Intervention for Symptoms of Anxiety and Depression Among University Students: Open-Label, Pragmatic Trial
Source: JMIR Ment Health. 2021 May 27;8(5):e27400. doi: 10.2196/27400 (PMC8193479; doi:10.2196/27400)
Supplement: Multimedia Appendix 1 [file mental_v8i5e27400_app1.docx]

**Supplementary Tables**

| **Table S1**: Analysis of differences in sample characteristics among participants who completed the follow-up assessments and those lost to follow-up | | | | | | | | | |
| --- | --- | --- | --- | --- | --- | --- | --- | --- | --- |
|  | Followed-up (n=125) | | | Lost to follow up (n=33) | | |  | | ***P value*** |
|  | Mean | S.E. | S.D. | Mean | S.E. | S.D. |  |  |  |
| Female | 85.6% | 3.2% | 35.3% | 84.8% | 6.3% | 36.4% | X^~~2~~^(1)= | 0.11 | .86 |
| Age | 22.0 | 0.4 | 4.5 | 23.5 | 1.4 | 7.9 | U= | 1819.0 | .29 |
| Undergraduates | 78.4% | 3.7% | 41.3% | 66.7% | 8.3% | 47.9% | X^~~2~~^(1)= | 1.9 | .16 |
| Number of sessions attended | 6.8 | 0.2 | 2.6 | 4.8 | 0.6 | 3.2 | U= | 1320.5 | **.00** |

| **Table S2**: Analysis of differences in symptom severity between participants who were followed up and those lost to follow-up | | | | | | | |
| --- | --- | --- | --- | --- | --- | --- | --- |
|  |  | None | Mild | Moderate | Severe | X^2^(3) | ***P value*** |
| Symptoms of depression | Followed up | 20 | 43 | 55 | 7 | 3.91 | .27 |
|  | Lost to follow up | 3 | 8 | 18 | 4 |  |  |
| Symptoms of anxiety | Followed up | 31 | 37 | 52 | 5 | 4.24 | .24 |
|  | Lost to follow up | 3 | 10 | 18 | 2 |  |  |

| **Table S3**: Analysis of differences in sample characteristics among participants who completed at least one session of the intervention and those who did not | | | | | | | | | |
| --- | --- | --- | --- | --- | --- | --- | --- | --- | --- |
|  | Started intervention (n=158) | | | No intervention (n=17) | | |  | | ***P value*** |
|  | Mean | S.E. | S.D. | Mean | S.E. | S.D. |  |  |  |
| Female | 84.8% | 2.9% | 36.0% | 94.1% | 5.9% | 24.3% | X^~~2~~^(1)= | 1.09 | .297 |
| Age | 22.4 | 0.4 | 5.4 | 21.2 | 0.5 | 2.2 | U= | 1284.0 | .76 |
| Undergraduates | 75.95% | 3.41% | 42.9% | 88.2% | 8.1% | 33.2% | X^~~2~~^(1)= | 1.31 | .25 |

| **Table S4**: Analysis of differences in symptom severity between participants who completed at least one session of the intervention and those who did not | | | | | | | |
| --- | --- | --- | --- | --- | --- | --- | --- |
|  |  | None | Mild | Moderate | Severe | X^2^(3) | ***P value*** |
| Symptoms of depression | Started intervention (n=158) | 24 | 51 | 75 | 8 | 7.26 | .06 |
|  | No intervention (n=17) | 5 | 1 | 11 | 0 |  |  |
| Symptoms of anxiety | Started intervention (n=158) | 35 | 47 | 72 | 4 | 1.32 | .72 |
|  | No intervention (n=17) | 5 | 5 | 6 | 1 |  |  |

| **Table S5**: Prevalence of symptoms at baseline and follow-up among female students (n=107) | | | | | | | | | | | | | |
| --- | --- | --- | --- | --- | --- | --- | --- | --- | --- | --- | --- | --- | --- |
|  |  | **Threshold** | | **None^8^** | | **Mild^7^** | | **Moderate^6^** | | **Severe^5^** | | **X^2^(3)** | ***P value*** |
| **GAD^1^** | Baseline | 44.9% | (4.8%) | 26.2% | (4.3%) | 29.0% | (4.4%) | 22.4% | (4.1%) | 22.4% | (4.1%) | 23.5 | **<0.001** |
|  | Follow-up | 15.0% | (3.5%) | 45.8% | (4.8%) | 39.3% | (4.7%) | 8.4% | (2.7%) | 6.5% | (2.4%) |  |  |
|  | Absolute change in prevalence | 29.9% | |  |  |  |  | 14.0% | | 15.9% | |  |  |
|  | Proportional change in prevalence | 66.7% | |  |  |  |  | 62.5% | | 70.8% | |  |  |
|  |  | X^2^(1) | 20.9 |  |  |  |  |  |  |  |  |  |  |
|  |  |  | *P* **<0.001** |  |  |  |  |  |  |  |  |  |  |
| **MDE^2^** | Baseline | 51.4% | (4.9%) | 17.8% | (3.7%) | 30.8% | (4.5%) | 46.7% | (4.8%) | 4.7% | (2.0%) | 27.0 | **<0.001** |
|  | Follow-up | 18.7% | (3.8%) | 38.3% | (4.7%) | 43.0% | (4.8%) | 15.9% | (3.6%) | 2.8% | (1.6%) |  |  |
|  | Absolute change in prevalence | 32.7% | |  |  |  |  | 30.8% | | 1.9% | |  |  |
|  | Proportional change in prevalence | 63.6% | |  |  |  |  | 66.0% | | 40.0% | |  |  |
|  |  | X^2^(1) | 28.2 |  |  |  |  |  |  |  |  |  |  |
|  |  |  | *P* **<0.001** |  |  |  |  |  |  |  |  |  |  |
| **GAD and MDE ^4^** | Baseline | 48.6% | (4.9%) | 20.6% | (3.9%) | 30.8% | (4.5%) | 27.1% | (4.3%) | 21.5% | (4.0%) | 32.0 | 5.03 |
|  | Follow-up | 14.0% | (3.4%) | 43.0% | (4.8%) | 43.0% | (4.8%) | 10.3% | (2.9%) | 3.7% | (1.8%) |  |  |
|  | Absolute change in prevalence | 34.6% | |  |  |  |  | 16.8% | | 17.8% | |  |  |
|  | Proportional change in prevalence | 71.2% | |  |  |  |  | 62.1% | | 82.6% | |  |  |
|  |  | X^2^(1) | 28.8 |  |  |  |  |  |  |  |  |  |  |
|  |  |  | *P* **<0.001** |  |  |  |  |  |  |  |  |  |  |

| **Table S6**: Prevalence of symptoms at baseline and follow-up among male students (n=18) | | | | | | | | | | | | | |
| --- | --- | --- | --- | --- | --- | --- | --- | --- | --- | --- | --- | --- | --- |
|  |  | **Threshold** | | **None^8^** | | **Mild^7^** | | **Moderate^6^** | | **Severe^5^** | | **X^2^(3)** | ***P value*** |
| **GAD^1^** | Baseline | 50.0% | (12.1%) | 16.7% | (9.0%) | 33.3% | (11.4%) | 44.4% | (12.1%) | 5.6% | (5.6%) | 6.1 | .11 |
|  | Follow-up | 27.8% | (10.9%) | 55.6% | (12.1%) | 16.7% | (9.0%) | 22.2% | (10.1%) | 5.6% | (5.6%) |  |  |
|  | Absolute change in prevalence | 22.2% | |  |  |  |  | 22.2% | | 0.0% | |  |  |
|  | Proportional change in prevalence | 44.4% | |  |  |  |  | 50.0% | | 0.0% | |  |  |
|  |  | X^2^(1) | n/a |  |  |  |  |  |  |  |  |  |  |
|  |  |  | *P* =0.29 |  |  |  |  |  |  |  |  |  |  |
| **MDE^2^** | Baseline | 38.9% | (11.8%) | 5.6% | (5.6%) | 55.6% | (12.1%) | 38.9% | (11.8%) | 0.0% | (0.0%) | n/a | n/a |
|  | Follow-up | 16.7% | (9.0%) | 33.3% | (11.4%) | 50.0% | (12.1%) | 16.7% | (9.0%) | 0.0% | (0.0%) |  |  |
|  | Absolute change in prevalence | 22.2% | |  |  |  |  | 22.2% | | 0.0% | |  |  |
|  | Proportional change in prevalence | 57.1% | |  |  |  |  | 57.1% | |  | |  | 1.0 |
|  |  | X^2^(1) | n/a |  |  |  |  |  |  |  |  |  |  |
|  |  |  | *P* =0.13 |  |  |  |  |  |  |  |  |  |  |
| **GAD and MDE ^4^** | Baseline | 50.0% | (12.1%) | 16.7% | (9.0%) | 33.3% | (11.4%) | 44.4% | (12.1%) | 5.6% | (5.6%) | 4.5 | .21 |
|  | Follow-up | 22.2% | (10.1%) | 44.4% | (12.1%) | 33.3% | (11.4%) | 16.7% | (9.0%) | 5.6% | (5.6%) |  |  |
|  | Absolute change in prevalence | 27.8% | |  |  |  |  | 27.8% | | 0.0% | |  |  |
|  | Proportional change in prevalence | 55.6% | |  |  |  |  | 62.5% | |  | |  |  |
|  |  | X^2^(1) | n/a |  |  |  |  |  |  |  |  |  |  |
|  |  |  | *P* =.06 |  |  |  |  |  |  |  |  |  |  |

| **Table S7**: Satisfaction with treatment by severity of symptoms at baseline (n=125)   \|  \|  \| **Total sample**  % (S.E) \| \| **MDE and GAD^1^** \| \| \| \| \| \| \| \| \| \| \| --- \| --- \| --- \| --- \| --- \| --- \| --- \| --- \| --- \| --- \| --- \| --- \| --- \| --- \| \|  \|  \| No symptoms ^2^  % (S.E) \| \| Mild Symptoms^3^  % (S.E) \| \| Moderate symptoms ^4^  ^% (S.E)^ \| \| Severe symptoms ^5^  % (S.E) \| \| X^2^(3) \| P \| \| Quality of the intervention \| Excellent \| 56,1% \| (4,5%) \| 45,5% \| (10,9%) \| 63,5% \| (6,7%) \| 56,7% \| (9,2%) \| 47,4% \| (11,8%) \| 2,7 \| 0,43 \| \| Good \| 35,0% \| (4,3%) \| 45,5% \| (10,9%) \| 30,8% \| (6,5%) \| 26,7% \| (8,2%) \| 47,4% \| (11,8%) \| 3,7 \| 0,30 \| \| Fair \| 6,5% \| (2,2%) \| 9,1% \| (6,3%) \| 3,8% \| (2,7%) \| 10,0% \| (5,6%) \| 5,3% \| (5,3%) \| 1,5 \| 0,68 \| \| Poor \| 2,4% \| (1,4%) \| 0,0% \| (0,0%) \| 1,9% \| (1,9%) \| 6,7% \| (4,6%) \| 0,0% \| (0,0%) \| 3,3 \| 0,34 \| \| Did you receive the kind of help that you wanted? \| Yes, definitely \| 39,8% \| (4,4%) \| 40,9% \| (10,7%) \| 42,3% \| (6,9%) \| 33,3% \| (8,8%) \| 42,1% \| (11,6%) \| 0,7 \| 0,87 \| \| Yes, generally \| 46,3% \| (4,5%) \| 45,5% \| (10,9%) \| 50,0% \| (7,0%) \| 43,3% \| (9,2%) \| 42,1% \| (11,6%) \| 0,5 \| 0,91 \| \| No, not really \| 12,2% \| (3,0%) \| 13,6% \| (7,5%) \| 7,7% \| (3,7%) \| 16,7% \| (6,9%) \| 15,8% \| (8,6%) \| 1,8 \| 0,61 \| \| No, definitely not \| 1,6% \| (1,1%) \| 0,0% \| (0,0%) \| 0,0% \| (0,0%) \| 6,7% \| (4,6%) \| 0,0% \| (0,0%) \| 6,3 \| 0,10 \| \| Satisfaction with amount of help received \| Very satisfied \| 48.4% \| (4,5%) \| 36,4% \| (10,5%) \| 63,5% \| (6,7%) \| 34.5% \| (8,8%) \| 42,1% \| (11,6%) \| **9,0** \| **0,03** \| \| Mostly satisfied \| 38.5% \| (4,4%) \| 50,0% \| (10,9%) \| 25,0% \| (6,1%) \| 48.3% \| (9,3%) \| 47,4% \| (11,8%) \| 6,7 \| 0,08 \| \| Indifferent or mildly dissatisfied \| 10.7% \| (2,8%) \| 9,1% \| (6,3%) \| 9,6% \| (4,1%) \| 13.8% \| (6,3%) \| 10,5% \| (7,2%) \| 0,3 \| 0,95 \| \| Quite dissatisfied \| 2.5% \| (1,4%) \| 4,5% \| (4,5%) \| 1,9% \| (1,9%) \| 3.4% \| (3,3%) \| 0,0% \| (0,0%) \| 1,0 \| 0,79 \| \| Intervention helped to deal more effectively with problems \| Yes, they helped a great deal \| 48,0% \| (4,5%) \| 31.8% \| (0,098) \| 55,8% \| (0,070) \| 46,7% \| (0,093) \| 47,4% \| (0,118) \| 3,590 \| 0,31 \| \| Yes, they helped \| 41,5% \| (4,4%) \| 54.5% \| (0,106) \| 38,5% \| (0,068) \| 36,7% \| (0,089) \| 42,1% \| (0,116) \| 2,032 \| 0,57 \| \| No, they really didn’t help \| 10,6% \| (2,8%) \| 13.6% \| (0,072) \| 5,8% \| (0,033) \| 16,7% \| (0,069) \| 10,5% \| (0,072) \| 2,667 \| 0,45 \| \| No, they made things worse \| 0,0% \| (0,0%) \| 0,0% \| (0,000) \| 0,0% \| (0,000) \| 0,0% \| (0,000) \| 0,0% \| (0,000) \| n/a \|  \| \| Extent to which intervention met needs \| Almost all of my needs have been met \| 25,2% \| (3,9%) \| 18,2% \| (0,084) \| 28,8% \| (0,063) \| 26,7% \| (0,082) \| 21,1% \| (0,096) \| 1,149 \| 0,77 \| \| Most of my needs have been met \| 48,8% \| (4,5%) \| 45,5% \| (0,109) \| 51,9% \| (0,070) \| 40,0% \| (0,091) \| 57,9% \| (0,116) \| 1,116 \| 0,57 \| \| Only a few of my needs have been met \| 22,0% \| (3,7%) \| 27,3% \| (0,097) \| 19,2% \| (0,055) \| 23,3% \| (0,079) \| 21,1% \| (0,096) \| 0,631 \| 0,89 \| \| None of my needs have been met \| 4,1% \| (1,8%) \| 9,1% \| (0,063) \| 0,0% \| (0,000) \| 10,0% \| (0,056) \| 0,0% \| (0,000) \| 7,143 \| 0,07 \| \| Likelihood recommend the intervention to a friend \| Yes, definitely \| 62,6% \| (4,4%) \| 59,1% \| (0,107) \| 69,2% \| (0,065) \| 56,7% \| (0,092) \| 57,9% \| (0,116) \| 1,723 \| 0,63 \| \| Yes, generally \| 32,5% \| (4,2%) \| 40,9% \| (0,107) \| 25,0% \| (0,061) \| 33,3% \| (0,088) \| 42,1% \| (0,116) \| 2,850 \| 0,42 \| \| No, not really \| 3,3% \| (1,6%) \| 0,0% \| (0,000) \| 3,8% \| (0,027) \| 6,7% \| (0,046) \| 0,0% \| (0,000) \| 2,548 \| 0,47 \| \| No, definitely not \| 1,6% \| (1,1%) \| 0,0% \| (0,000) \| 1,9% \| (0,019) \| 3,3% \| (0,033) \| 0,0% \| (0,000) \| 1,253 \| 0,74 \| \| Overall satisfaction \| Very satisfied \| 56,5% \| (4,5%) \| 40.9% \| (0,104) \| 69,2% \| (0,065) \| 46,7% \| (0,093) \| 57,9% \| (0,116) \| 6,807 \| 0,08 \| \| Mostly satisfied \| 33,9% \| (4,3%) \| 50.0% \| (0,106) \| 23,1% \| (0,059) \| 40,0% \| (0,091) \| 36,8% \| (0,114) \| 5,811 \| 0,12 \| \| Indifferent or mildly dissatisfied \| 8,1% \| (2,5%) \| 9.1% \| (0,060) \| 7,7% \| (0,037) \| 10,0% \| (0,056) \| 5,3% \| (0,053) \| 0,390 \| 0,94 \| \| Quite dissatisfied \| 0,0% \| (0,0%) \| 0,0% \| (0,000) \| 0,0% \| (0,000) \| 0,0% \| (0,000) \| 0,0% \| (0,000) \| n/a \|  \| \| 1 PHQ-9 score + GAD-7 score \| \| \| \| \| \| \| \| \| \| \| \| \| \| \| 2 PHQ-9 score + GAD-7 score < 10 \| \| \| \| \| \| \| \| \| \| \| \| \| \| \| 3 PHQ-9 score + GAD-7 score = 10 to 19 \| \| \| \| \| \| \| \| \| \| \| \| \| \| \| 4 PHQ-9 score + GAD-7 score = 20 - 29 \| \| \| \| \| \| \| \| \| \| \| \| \| \| \| 5 PHQ-9 score + GAD-7 score =>29 \| \| \| \| \| \| \| \| \| \| \| \| \| \| \| *** p <0.05** \|  \|  \|  \|  \|  \|  \|  \|  \|  \|  \|  \|  \|  \| |
| --- | --- | --- | --- | --- | --- | --- | --- | --- | --- | --- | --- | --- | --- | --- | --- | --- | --- | --- | --- | --- | --- | --- | --- | --- | --- | --- | --- | --- | --- | --- | --- | --- | --- | --- | --- | --- | --- | --- | --- | --- | --- | --- | --- | --- | --- | --- | --- | --- | --- | --- | --- | --- | --- | --- | --- | --- | --- | --- | --- | --- | --- | --- | --- | --- | --- | --- | --- | --- | --- | --- | --- | --- | --- | --- | --- | --- | --- | --- | --- | --- | --- | --- | --- | --- | --- | --- | --- | --- | --- | --- | --- | --- | --- | --- | --- | --- | --- | --- | --- | --- | --- | --- | --- | --- | --- | --- | --- | --- | --- | --- | --- | --- | --- | --- | --- | --- | --- | --- | --- | --- | --- | --- | --- | --- | --- | --- | --- | --- | --- | --- | --- | --- | --- | --- | --- | --- | --- | --- | --- | --- | --- | --- | --- | --- | --- | --- | --- | --- | --- | --- | --- | --- | --- | --- | --- | --- | --- | --- | --- | --- | --- | --- | --- | --- | --- | --- | --- | --- | --- | --- | --- | --- | --- | --- | --- | --- | --- | --- | --- | --- | --- | --- | --- | --- | --- | --- | --- | --- | --- | --- | --- | --- | --- | --- | --- | --- | --- | --- | --- | --- | --- | --- | --- | --- | --- | --- | --- | --- | --- | --- | --- | --- | --- | --- | --- | --- | --- | --- | --- | --- | --- | --- | --- | --- | --- | --- | --- | --- | --- | --- | --- | --- | --- | --- | --- | --- | --- | --- | --- | --- | --- | --- | --- | --- | --- | --- | --- | --- | --- | --- | --- | --- | --- | --- | --- | --- | --- | --- | --- | --- | --- | --- | --- | --- | --- | --- | --- | --- | --- | --- | --- | --- | --- | --- | --- | --- | --- | --- | --- | --- | --- | --- | --- | --- | --- | --- | --- | --- | --- | --- | --- | --- | --- | --- | --- | --- | --- | --- | --- | --- | --- | --- | --- | --- | --- | --- | --- | --- | --- | --- | --- | --- | --- | --- | --- | --- | --- | --- | --- | --- | --- | --- | --- | --- | --- | --- | --- | --- | --- | --- | --- | --- | --- | --- | --- | --- | --- | --- | --- | --- | --- | --- | --- | --- | --- | --- | --- | --- | --- | --- | --- | --- | --- | --- | --- | --- | --- | --- | --- | --- | --- | --- | --- | --- | --- | --- | --- | --- | --- | --- | --- | --- | --- | --- | --- | --- | --- | --- | --- | --- | --- | --- | --- | --- | --- | --- | --- | --- | --- | --- | --- | --- | --- | --- | --- | --- | --- | --- | --- | --- | --- | --- | --- | --- | --- | --- | --- | --- | --- | --- | --- | --- | --- | --- | --- | --- | --- | --- | --- | --- | --- | --- | --- | --- | --- | --- | --- | --- | --- | --- | --- | --- | --- | --- | --- | --- | --- | --- | --- | --- | --- | --- | --- | --- | --- | --- | --- | --- | --- | --- | --- | --- | --- | --- | --- | --- | --- | --- | --- | --- | --- | --- | --- | --- | --- | --- | --- | --- | --- | --- | --- | --- | --- | --- | --- | --- | --- | --- | --- | --- | --- |

| **Table S8** Multivariate regression analysis of predictors of satisfaction (total satisfaction score) | | | |
| --- | --- | --- | --- |
|  | Beta | 96%CI | ***P value*** |
| Female gender | 0.2 | (0.6-4.7) | .01 |
| Age | 0.1 | (-0.1-0.2) | .53 |
| Number of sessions | -0.2 | (-0.5-0.0) | .08 |
| Baseline PHQ score | -0.1 | (-0.2-0.1) | .65 |
| Baseline GAD score | 0.1 | (-0.1-0.2) | .44 |
| Proportional improvement in symptoms | -0.1 | (-1.3-0.7) | .57 |
|  |  | F(6)=1.818 |  |
|  |  | *P*=.10 |  |
